# Management of Advanced Heart Failure in Children with Cancer Therapy-Related Cardiac Dysfunction

**DOI:** 10.3390/children8100872

**Published:** 2021-09-30

**Authors:** Hari P. Tunuguntla, Kriti Puri, Susan W. Denfield

**Affiliations:** 1 Lillie Frank Abercrombie Section of Cardiology, Department of Pediatrics, Baylor College of Medicine, Texas Children’s Hospital, Houston, TX 77030, USA; tunugunt@bcm.edu (H.P.T.); kriti.puri@bcm.edu (K.P.); 2 Section of Critical Care Medicine, Department of Pediatrics, Baylor College of Medicine, Texas Children’s Hospital, Houston, TX 77030, USA

**Keywords:** chemotherapy-induced cardiomyopathy, ventricular assist device, heart transplant

## Abstract

The evolution of cancer therapies has led to marked improvement in survival of those affected by childhood malignancies, while also increasing the recognition of early and late toxicities associated with cancer therapies. Cardiotoxicity can include cardiomyopathy/heart failure, coronary artery disease, stroke, pericardial disease, arrhythmias, and valvular and vascular dysfunction as a result of exposure to chemotherapy and/or radiation. Anthracyclines remain the most common cause of chemotherapy-induced cardiomyopathy (CCM) with varying clinical presentations including: acute, early onset, and late-onset. Many individuals develop cardiac dysfunction over the long-term, ranging from subclinical cardiac dysfunction to end-stage symptomatic heart failure. The focus of this review is on characterization of symptomatic heart failure in children with cancer therapy-related cardiac dysfunction (CTRCD) primarily due to CCM and utilization of advanced heart failure therapies, including ventricular assist device (VAD) support and heart transplantation, with consideration of unique patient-related factors.

## 1. Introduction

The evolution of cancer therapies has led to marked improvement in survival of those affected by childhood malignancies. The five-year survival in children and adolescents with cancer ranged from 58–68% in the 1970s to 84–85% during the 2010–2016 time frame [1,2]. This trend has also resulted in increased recognition of the early and late toxicities associated with cancer therapies. The cardiotoxic effects of anti-cancer therapies are broad and variable in onset including cardiomyopathy/heart failure, coronary artery disease, stroke, pericardial disease, arrhythmias, and valvular and vascular dysfunction [3,4,5]. The Childhood Cancer Survivor Study (CCSS), a study following a cohort of survivors who were treated from 1970–1986 and had survived at least five years after treatment, has shown that survivors were found to have an increased relative risk of a chronic health condition compared to their siblings of 3.3 (95% CI, 3.0–3.5) [6]. A sub-analysis of the CCSS population focusing on cardiovascular health reported that cancer survivors were significantly more likely to have cardiovascular complications, including a higher frequency of congestive heart failure (1.7% versus 0.2% among siblings), valvular abnormalities (1.6% versus 0.5%) and pericardial disease (1.3% versus 0.3%) [7]. There is a spectrum of cardiac dysfunction ranging from subclinical cardiac dysfunction to end-stage symptomatic heart failure. In this review, we will characterize at risk populations and progression of anthracycline cardiotoxicity, various presentations of symptomatic heart failure in children with cancer therapy-related cardiac dysfunction (CTRCD) primarily due to chemotherapy-induced cardiomyopathy (CCM), and unique considerations in utilizing advanced heart failure therapies in this population.

## 2. Risk Factors for Cardiac Therapy-Related Cardiac Dysfunction

An understanding of risk factors for development of CTRCD is important in identifying individuals who are at increased risk for development of heart failure. Chemotherapeutic agents including anthracyclines, tyrosine kinase inhibitors, monoclonal antibodies, and immune checkpoint inhibitors can all have variable cardiotoxic side effects. Radiation therapy can also result in cardiotoxicity including pericarditis, cardiomyopathy (more commonly with a restrictive phenotype than dilated), coronary artery disease (which may lead to acute myocardial infarction), valvular disease, and conduction system abnormalities [4,5,8].

Among chemotherapeutic agents, anthracyclines are commonly utilized in the treatment of hematological and solid tumors in children and adults and are most frequently linked to cardiac dysfunction [4,9]. The frequency of subclinical cardiac dysfunction among childhood cancer survivors of pediatric malignancies varies widely, with an estimated 25–50% of all survivors having subclinical cardiac dysfunction within 20 years after therapy [4,10,11,12,13]. Studies have also shown that the cumulative dose, dose rate, dosing schedule of anthracyclines, and concomitant use with other cardiotoxic therapies can further impact risk for developing cardiotoxicity. Other non-modifiable risk factors for development of cardiac dysfunction include female sex and young age < four years at time of treatment [9,13,14,15,16,17,18].

There are two major classifications of cardiotoxicity, which can elucidate whether there may be potential for reversible cardiac changes [8,19]. Anthracyclines are the prototypical drug related to Type 1 cardiotoxicity, and are associated with histopathologic changes in the cardiomyocyte ultrastructure including myocyte necrosis and cell death. This type of damage is dose dependent, progressive, and irreversible and can manifest as cardiac dysfunction many years after therapy. Type 2 cardiotoxicity, which can occur from agents such as trastuzumab, does not cause ultrastructural change in the myocardium, resulting in a higher likelihood of recovery to baseline cardiac function after discontinuation of therapy. Many chemotherapeutic agents, however, cannot be classified into one of these distinct categories [8,19].

The onset of cardiotoxicity related to anthracyclines can be acute, defined as within a week of anthracycline therapy; subacute, occurring within a year of anthracycline therapy; or late, defined as >one year after anthracycline therapy. Acute cardiotoxicity may present as arrhythmias or ECG abnormalities, transient depression in myocardial contractility, or can mimic heart failure related to myocarditis or pericarditis in the setting of high doses. It generally resolves after removal of the offending agent. However, subacute (>one week and <one year from exposure) and late onset (>one year from exposure) cardiotoxicity are more common clinical presentations. These types of cardiotoxicities are progressive in nature and can be associated with echocardiogram features of dilated or restrictive cardiomyopathy as well as arrhythmias [9,14,15,16,17,18]. The progression of cardiomyopathy was best described by Lipshultz et al. (2005). In this study of pediatric acute lymphoblastic leukemia patients, the echocardiograms initially demonstrated features of dilated cardiomyopathy with reduced left ventricle (LV) fractional shortening (FS) with reduced contractility and LV dilation with wall thinning. Over time, there was further progression to a phenotype of restrictive cardiomyopathy with normal to reduced LV dimensions and reduced LV wall thickness, LV FS, and contractility. These findings were particularly evident in individuals with a younger age at cancer diagnosis and longer time since cancer diagnosis [11,18].

## 3. Advanced Heart Failure Therapies

Early detection and treatment of subclinical cardiac dysfunction is essential to prevent or delay irreversible cardiac injury and advanced heart failure. The American Heart Association and American College of Cardiology provide a framework for characterizing the spectrum of heart failure which can further guide management. This includes four stages of heart failure: Stage A—patients at risk for cardiac dysfunction; Stage B—patients with cardiac dysfunction without symptoms; Stage C—patients with cardiac dysfunction with past or current symptoms; Stage D—patients with heart failure symptoms refractory to medical therapy [20]. This framework can also be applied to the pediatric population.

Preventative strategies to mitigate cardiotoxicity can be employed, in some circumstances, even prior to the development of cardiac dysfunction. When using anthracyclines, this may include dose adjustment, modifying administration as a continuous infusion to avoid high serum concentrations, utilizing anthracycline analogues, and use of an iron-chelating agent such as dexrazoxane to prevent reactive oxygen species related cardiotoxicity caused by anthracyclines [14,21,22].

Guideline-directed medical therapies for heart failure with reduced ejection fraction are commonly utilized for patients with Stage B or Stage C heart failure. The rationale for use of standard heart failure medications such as angiotensin-converting enzyme (ACE) inhibitors and beta-blockers is largely extrapolated from the adult experience with limited pediatric data, especially specific to pediatric patients with CCM. ACE inhibitors have been shown to reduce LV wall thickness and LV afterload while improving cardiac function in many cardiac pathologies including CCM [23,24,25]. Beta-blockers have also been shown to improve hemodynamics as well as ventricular function in adults with both ischemic and non-ischemic cardiomyopathy, likely through reversal of adrenergic mediated cardiac dysfunction and remodeling [26,27]. Cardinale et al. demonstrated that oral reverse remodeling therapies such as angiotensin-converting enzyme (ACE) inhibitors and beta-blockers were beneficial in adults with anthracycline related cardiomyopathy. However, this study also demonstrated that there were fewer responders to therapy with increased time to initiation of treatment from the end of chemotherapy. Additionally, recovery of LV function was not seen beyond six months of treatment [25].

Additional studies have demonstrated that there is limited efficacy of ACE inhibitors in limiting the progression of ventricular dysfunction in childhood cancer survivors [24,28]. Lipshultz et al. demonstrated that afterload reduction with ACE inhibitors improved structure and function in the short term in long-term survivors of childhood cancer; however, these improvements were lost after six to 10 years and cardiac disease can further progress [24]. Multiple adult studies show potential for the role of beta-blockers in preventing cardiac dysfunction [29,30,31]. Alternative therapies such as stem cell therapy for treatment of CCM remain in trial and under investigational use [32]. When heart failure progresses to the point of requiring care in the intensive care unit (ICU), the usual armamentarium of inotropic therapies is utilized. Advanced heart failure (Stage D) may ensue in some individuals warranting treatment with cardiac resynchronization therapy, mechanical circulatory support, and/or cardiac transplantation. 

The number of patients with advanced heart failure among cancer survivors is not clearly established in the adult or pediatric population. In a review of two adult registries including United Network of Organ Sharing (UNOS) and Interagency Registry for Mechanically-Assisted Circulatory Support (INTERMACS), it was estimated that CCM was the attributable cause for advanced heart failure in 0.5–2.5% of patients [33,34,35]. While this is a relatively low proportion of the adult heart failure population, these registries cannot determine the percent of adult cancer survivors at risk. The 0.5–2.5% of patients is likely an underestimate as the diagnosis of chemotherapy-induced cardiomyopathy is often encompassed within the diagnosis of dilated cardiomyopathy in many registries. Moreover, individuals with advanced heart failure due to CTRCD may not be identified if they are not candidates for advanced heart failure treatments. 

The complexity of cancer therapies, comorbid conditions of patients with CTRCD, as well as the potential for relapse, require special consideration when considering timing and implementation of more advanced heart failure therapies such as VAD and cardiac transplant. Usually, VAD and transplant are reserved for those who are in complete remission and considered to be at low risk for recurrence, typically three to five years after the end of cancer therapies depending on the cancer. Ventricular assist device therapy may also need to be considered in the setting of ongoing chemotherapy as a bridge to recovery of function or as a bridge to eventual cardiac transplant when deemed cancer free for a sufficient period of time. This requires a very complex multi-team approach when considering the potential side effects of chemotherapy in regards to the possible development of pancytopenias which complicate infection issues and bleeding and clotting risks in a patient on an LVAD. Families also have to be aware that, when facing end of life issues in these complex patients, the LVAD may have to be turned off to allow the patient to die. Therefore, it is extremely important for the heart failure/transplant team to partner with the patient’s oncologist when considering these advanced therapies [36,37]. 

We will review the current literature evaluating epidemiology and outcomes of CRT, VAD and transplant therapy in treatment of advanced heart failure due to CCM and two previously reported cases from our center in children with advanced heart failure due to cancer therapies highlighting the application of VAD support and heart transplant.

## 4. Cardiac Resynchronization Therapy (CRT)

According to the European Society of Cardiology as well as American College of Cardiology/American Heart Association Guidelines, an implantable cardioverter-defibrillator (ICD) can prevent mortality due to sudden arrhythmic death and is recommended for individuals with symptomatic HF and left ventricular ejection fraction (LVEF) < 35% despite >three months of treatment with optimal pharmacological therapy. Cardiac resynchronization therapy (CRT) can reduce mortality while also improving cardiac function and symptoms in select adult patients with symptomatic heart failure. CRT is recommended for adult patients with symptomatic heart failure despite medical therapy in sinus rhythm with LVEF < 35%, QRS duration >150 msec, and left bundle branch block (LBBB) QRS morphology. CRT can also be considered for symptomatic patients with LVEF < 35% with intermediate QRS duration and/or non-LBBB QRS morphology [38,39,40,41].

The application and outcome of these therapies in the pediatric population has been variable, likely due in part to the heterogeneity of causes of pediatric heart failure [42]. According to the 2014 ISHLT pediatric heart failure guidelines, CRT can be useful (Class IIa) in pediatric patients with symptomatic heart failure, systemic left ventricle, EF < 35%, and ECG characteristics including left bundle branch block and prolonged QRS duration for age. ICD therapy can be considered (Class IIa or IIb indication) in pediatric patients with dilated cardiomyopathy who have LVEF < 35% without significant risk factors precluding the procedure itself. ICD is clearly indicated (Class I indication) in pediatric survivors of cardiac arrest after evaluation for reversible or treatable causes for the event [43].

CRT has been shown to be underutilized in adult cancer survivors with both ischemic and non-ischemic heart failure, despite meeting clinical indications for this therapy. In a review of the INTERMACS registry, patients with CCM were less likely to be implanted with an ICD compared to other forms of cardiomyopathy (CCM 66% vs. non-ischemic cardiomyopathy 77%; *p* = 0.03 vs. ischemic cardiomyopathy 77%; *p* = 0.03). It is postulated that this could be in part related to the acuity of heart failure and comorbidities at the time of clinical presentation [19,33,34,35]. The experience with CRT has largely been limited to adult cancer survivors. A retrospective study at the Mayo Clinic, including 29 patients with CTRCD who underwent CRT showed improvement in echo parameters within six to 18 months including increased LV EF and decreased LV diastolic and systolic dimensions [44]. Similar findings were seen in the MADIT-CHIC (Multicenter Automatic Defibrillator Implantation Trial–Chemotherapy-Induced Cardiomyopathy) study, an un-controlled prospective cohort study. This study included 30 patients with chemotherapy-induced cardiomyopathy from 12 centers who demonstrated a significant improvement in LV EF and LV dimensions at six months post intervention [19,33,45]. The pediatric experience with CRT for chemotherapy-induced cardiomyopathy is limited to case reports. Jones et al. reported successful use of CRT in a nine-year-old girl with acute myeloid leukemia (AML) and acute doxorubicin induced cardiomyopathy who developed severely depressed LV function (EF 22%) and inotrope dependence with normal QRS duration and echo findings of intraventricular dyssynchrony. After implementation of CRT, the patient was able to wean off ventilatory and inotropic support within a week and transitioned to oral heart failure therapies with gradual improvement in LV EF to 55% over a year [46]. There is an ongoing need to better characterize which pediatric patients may benefit from CRT, as adult criteria may not be sufficient. 

## 5. Mechanical Circulatory Support

Although orthotopic heart transplant (OHT) remains the ultimate therapy for treatment of advanced heart failure, patients with CTRCD may not be suitable candidates if there has been inadequate time since treatment of their malignancy, treatment of the malignancy is ongoing, or if there is restrictive physiology with high pulmonary vascular resistance. In such circumstances, durable mechanical circulatory support (MCS) with a left ventricular assist device (LVAD) may be the most ideal treatment option as a bridge to decision (BTD), destination therapy (DT), or less commonly to recovery. Individuals with cardiac dysfunction primarily related to radiation therapy require special surgical considerations for MCS or heart transplant due to increased risk for fibrosis, which can complicate sternal entry and result in a higher likelihood for bleeding and infection [33,36,47,48].

Even in individuals who are candidates for heart transplant, LVAD therapy can allow for improved hemodynamic support as well as physical and nutritional rehabilitation while awaiting transplant and also provide an opportunity to observe for potential myocardial recovery and cancer recurrence. In a survey focused on decision making regarding VAD therapy for various pediatric populations, there was variability on whether VAD therapy would be considered for patients with CTRCD across centers depending on the proximity to cancer illness, need for ongoing chemotherapy, and overall prognosis [49]. This may reflect the evolving nature of pediatric MCS support in utilizing VADs in more complex patient populations along with a limited understanding of the efficacy of these therapies on how they may impact long-term survival and quality of life, especially in the setting of other high-risk comorbidities.

Oliveira et al. reviewed the adult INTERMACS registry from 2006–2011. Of 3812 MCS patients, 75 patients (2%) had chemotherapy-induced cardiomyopathy (CCM). Pre-implant characteristics and outcomes post implant were compared between the CCM, ischemic CM (ICM), and non-ischemic CM (NICM) groups. Within the CCM cohort, half (52%) of the patients had a history of breast cancer and 33% had history of lymphoma or other hematologic malignancy. There was a higher prevalence of females in the CCM group (72% vs. 24% and 13%, respectively). The CCM group was younger compared to the ICM (mean age 53 vs. 60 years old; *p* < 0.001), had lower BMI compared to the ICM and NICM groups and had fewer comorbid conditions such as hypertension, diabetes, and obesity. Device strategy was more likely to be DT (33%) in the CCM group [34]. 

Notably, there was significantly more RV dysfunction and severe tricuspid regurgitation in the CCM group. Additionally, patients with CCM had a significantly higher need for RVAD (19%, 14/75) either at time of VAD implant or post LVAD implant. Concomitant surgery (48%) at the time of VAD implant including tricuspid valve repair (n = 11, 15%) was also more common in the CCM group. In comparing adverse outcomes among the groups, there was an increased risk of bleeding in the CCM group. However, patients with CCM who underwent LVAD had comparable survival to other MCS groups. Within the CCM group, those who had biventricular assist device (BIVAD) support had worse outcomes with 43% mortality (6/14) compared to 20% (12/61) in the LVAD-only group [34]. 

The increased risk of RV failure with MCS use in CCM is noteworthy, and has been demonstrated in other studies as well, including an ISHLT registry analysis [50]. This highlights the unique pattern of dysfunction with CCM, which can impact the function of both ventricles. Additional mechanisms for RV failure may be late recognition of left sided heart failure as well as high pulmonary vascular resistance. Understanding the degree of RV failure when considering MCS is important in instituting early RVAD support and perhaps even considering alternate forms of MCS such as a total artificial heart in certain circumstances. An appreciation for these added complexities and how it may impact the overall morbidity for the patient is essential especially when pursuing DT VAD [19,33,34,35,36,50]. 

Overall, there is less experience in children on the use of VAD support for CCM. Table 1 summarizes a literature review of VAD therapy in children with CCM.

**Figure 1:**
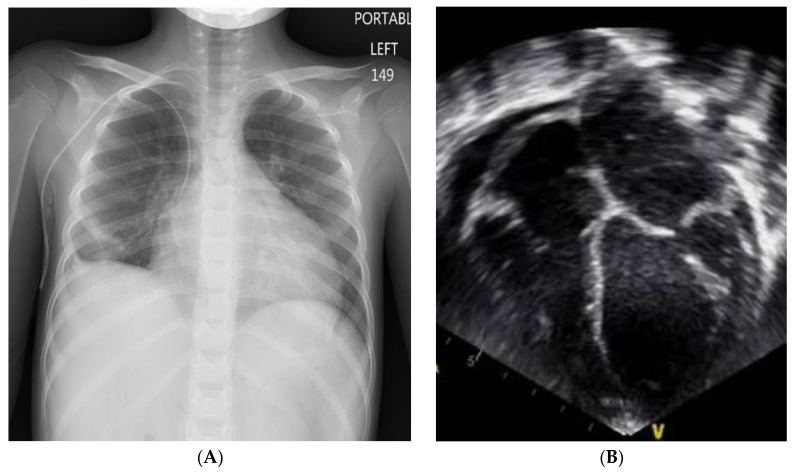
Myocardial recovery after VAD implant. (**A**) Chest x-ray image prior to VAD implant showing cardiomegaly. (**B**) Prior to VAD implant, trans-thoracic echocardiogram in the apical four-chamber view showing a moderately dilated left atrium (LA) and left ventricle (LV) with ventricular wall thinning and moderately depressed systolic function with a shortening fraction of 16% and calculated LVEF of 38%.

**Figure 2:**
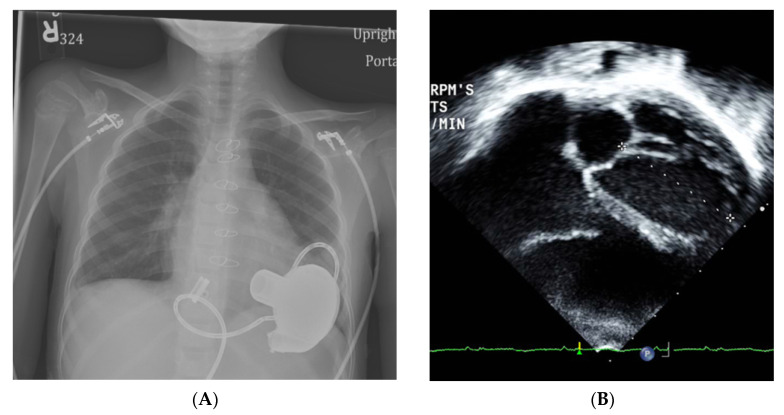
Myocardial recovery after VAD implant. (**A**) Chest-X-ray ~eight months post VAD implant showing resolution of cardiomegaly. (**B**) Post VAD implant, trans-thoracic echocardiogram in the apical four-chamber view showing normal LV dimensions with mildly depressed systolic function, LVEF 51%.

Early experience with VAD therapy was reported by the German Heart Institute Berlin, wherein two of five patients with chemotherapy-induced cardiomyopathy were successfully bridged to heart transplant with biventricular Berlin support after four to seven weeks [51]. Myocardial recovery after durable VAD implant in patients with CCM has been described in both adult and pediatrics, with the latter largely limited to case reports. Schweiger et al. published a case report describing the use of a HeartWare™ VAD in an eight-year-old patient with a body surface area of 0.97 m^2^ who had severe acute anthracycline-induced cardiomyopathy with successful explantation after 149 days of support [53,54]. Freilich et reported a 16-year-old girl in remission from lymphoma who developed cardiogenic shock due to anthracycline cardiomyopathy requiring central extracorporeal membrane oxygenation support initially but transitioned to a LVAD. The patient showed recovery of cardiac function at nine months post implant and was successfully explanted at 1 year after implant [52]. At our center, seven pediatric patients with durable VAD underwent a trial of explant with four of the seven remaining explanted and without transplant. Of these four patients, two had a diagnosis of CCM as etiology of their heart failure [52]. Figure 1 and Figure 2 demonstrate the effect of VAD therapy on LV decompression and associated recovery of ventricular function in a patient with CCM who underwent VAD explant [57]. Although predictors of myocardial recovery are not well understood, early implementation of LVAD therapy for decompensated heart failure combined with optimal use of neurohormonal blockade and other reverse remodeling therapies is likely to be instrumental in preventing irreversible cardiac damage from chemotherapeutic agents.

## 6. Heart Transplant

As patients with CCM have a history of malignancy, the risk for recurrence or development of post-transplant lymphoproliferative disorder (PTLD) or other de novo neoplasms must be considered given the increased risk of PTLD and other neoplasms in the immunosuppressed transplant patient. Historically, a history of solid organ or blood malignancy within five years would be a contraindication for heart transplant. However, this has evolved into a more individualized risk stratification in collaboration with oncology specialists to best understand optimal time for transplant eligibility [19,33,37,50].

Overall, both adult and pediatric studies have shown comparable long-term outcomes in patients with CCM compared to other cardiomyopathy patients. Specifically, Oliveira et al. reviewed the International Society of Heart and Lung Transplantation (ISHLT) Registry from 2000–2008 and found 232 patients with CCM. Survival at one-, two-, and five-years was similar between the CCM compared NI CM group (86% vs. 87%, 79% vs. 81%, and 71% vs. 74%; *p* = 0.19). The risk of cardiac allograft rejection in the 1st year after transplantation was lower in patients with CCM (28% vs. 38%; *p* = 0.03). Post-transplant infection rates were higher in the CCM group (22% vs. 14%; *p* = 0.04). There was a higher incidence of skin cancer in the CCM group, though malignancy recurrence or death from cancer was not increased compared to other cohorts [50]. A larger analysis of UNOS database from 1987–2011 was conducted by Lenneman et al. with similar findings of comparable survival [59].

Similar to the adult experience, most pediatric studies evaluating OHT in patients with CCM show favorable survival outcomes when compared to other cardiomyopathy cohorts. A study of the UNOS database with 7169 heart transplant recipients identified 1.5% (n = 107) of those transplanted had a history of childhood cancer. Post-transplant malignancy was higher in the cohort with a history of cancer before heart transplant compared to other recipients (13% versus 5.4%, *p* < 0.001). Post-transplant survival at one and five years was similar between the cancer and noncancer groups (90.6% and 80.3% in the cancer group vs. 84.4% and 73.8% in the noncancer group). This trend was maintained when limiting the comparison to patients with other forms of cardiomyopathy [60].

An analysis of the Pediatric Heart Transplant Society (PHTS) database from 1993–2014 compared patients with chemotherapy-induced cardiomyopathy with a propensity matched DCM cohort. Eighty children with CCM were listed for heart transplant and 78% (*n* = 62) underwent heart transplant, with 16% (*n* = 13) bridged to transplant with VAD. There was no significant difference in wait list mortality between the matched groups. Additionally, freedom from rejection and cardiac allograft vasculopathy did not differ between the groups. There were five post-transplant malignancies in four individuals, all related to PTLD and without recurrence of primary malignancy [56]. Multivariable analysis showed that earlier transplant era, VAD at listing, and female gender predicted worse post-transplant survival in the CCM group, rather than etiology of cardiomyopathy. Of note, although there was no difference in the frequency of induction therapy between the cohorts, there was an increased risk of serious infection in the CCM group resulting in a higher rate of post-transplant death due to infection (30% in the CCM cohort vs. 3.3% in the matched DCM cohort [*p*-value < 0.01]) [56]. Other smaller and earlier studies have also demonstrated rare recurrence of primary malignancy in some patients [61,62]. 

Overall, patients with CCM can have similar heart transplant graft survival to other cohorts. A comprehensive evaluation of all factors that can impact the immune system including oncologic diagnosis, timing and intensity of previous chemotherapy exposure, radiation exposure, and infection history may be particularly helpful in post-transplant management to minimize risk of infection and malignancy [37].

- Case 1:

A 14-year old female was diagnosed with AML. She received chemotherapy for approximately five months, including a total cumulative dose of 300 mg/m^2^ of daunorubicin (150 mg/m^2^ dose equivalent of doxorubicin). She developed heart failure around the third month of cancer treatment and therapy with oral reverse remodeling agents was initiated. Three months after completion of chemotherapy, she was admitted to the hospital with decompensated heart failure. Her LV ejection fraction (EF) was 10%, her right ventricular systolic function was moderately depressed, and her brain natriuretic peptide level was 1900 pg/mL. She could not be weaned from inotropic agents. She underwent LVAD placement with a HeartWare^®^ as a bridge to decision, as she had been in remission for only 3 months. She was followed as an outpatient on VAD support while also monitoring for cancer recurrence. She was noted to have early evidence of recovery of LV systolic function on echocardiogram three months after VAD support. Echocardiograms continued to show favorable signs of recovery with normalization of LV function and LV dimensions. She was continued on reverse remodeling agents and tolerated two pump-weaning tests well. She underwent LVAD explant 12 months after implantation (18 months after AML diagnosis). She is alive and doing well six years after completion of cancer treatment. She continues on treatment with oral reverse remodeling agents. Her echocardiograms have persistently shown mildly reduced left ventricular global longitudinal strain and normal ejection fraction [55].

- Case 2:

A newborn female was diagnosed with congenital AML. She received multi-drug chemotherapy with a total cumulative dose of 300 mg/m^2^ of daunorubicin (150 mg/m^2^ dose equivalent of doxorubicin) and 48 mg/m^2^ of mitoxantrone (480 mg/m^2^ dose equivalent of doxorubicin). The total cumulative equivalent dose of doxorubicin was 630 mg/m^2^. She developed worsening cardiac function 12 months after completion of chemotherapy. She was started on oral reverse remodeling agents and remained in cancer remission for 36 months. She developed symptomatic heart failure due to a mixed phenotype cardiomyopathy, with dilated and restrictive features and progressive pulmonary hypertension (PH) due to pre and post-capillary disease, indexed pulmonary vascular resistance (PVRi) 6.3 WUm^2^ reactive to 3.9 WUm^2^. She was evaluated and listed for heart transplant. She was subsequently admitted to the hospital for optimization of heart failure and pulmonary hypertension therapies. She was started on milrinone, but developed worsening heart failure and underwent HeartWare^®^ LVAD implantation (four years, 13 kg) in an effort to decompress the LV, improve pulmonary vascular resistance and allow escalation of PH therapies to improve her transplant candidacy. Her VAD course was complicated by two thromboembolic strokes requiring mechanical thrombectomy, which were likely multi-factorial in etiology, including a small LV cavity size and sinus node dysfunction/bradycardia secondary to right heart failure leading to lower VAD flows. However, she recovered well from the strokes without residual deficits and underwent heart transplantation six months after VAD implantation (~60 months after cancer diagnosis). She is alive one-year post heart transplant without evidence of rejection or malignancy recurrence [58].

## 7. Conclusions

There is an increasing awareness and understanding of the short- and long-term impacts of cancer therapies on the cardiovascular system. Advanced heart failure therapies including MCS and cardiac transplant can be successfully implemented in individuals with CTRCD. Patients with CCM undergoing MCS have similar survival to other groups but have increased risk of needing RVAD and bleeding in adult studies. Heart transplant survival is similar in patients with CCM compared to other forms of cardiomyopathy but there may be an increased risk of post-transplant infectious complications. Patient selection, optimal timing, and anticipation of the unique challenges that these patients pose are key elements to success with these interventions. Further multi-center studies are needed to better characterize the breadth of this population and their outcomes to increase timely utilization of advanced heart failure treatments, but more importantly to evaluate strategies that may mitigate the need for advanced heart failure therapies.

## Figures and Tables

**Table 1:** Literature review of ventricular assist device therapy in children with chemotherapy-induced cardiomyopathy.

Study	Patients (#) Age at Implant Weight, BSA	Cancer Diagnosis ^a^	Cumulative Anthracycline (Dose Equivalent of Doxorubicin)	Time of Implant (from Completion of Chemotherapy)	Type of Durable VAD	Support Duration	Outcome
Musci et al. 1997 [51]	2 patients, specific ages not stated	Cohort included: ALL, EWS, embryonal rhabdo-myosarcoma	435 mg/m^2^ (mean dose), (2 patients also had radiation therapy)	Not stated	Berlin EXCOR^®^ biventricular system	4 weeks, 7 weeks	Heart transplant ×2
Freilech et al. 2009 [52]	1 patient 16 years	Non-Hodgkin’s Lymphoma	400 mg/m^2^	3–4 months (8 months from diagnosis)	VentrAssist^TM^ LVAD (ECMO 6 d before)	1 year 13d	Recovery, explant
Schweiger et al. 2013, [53] Cavigelli et al. 2014 [54]	1 patient 8 years 25 kg, BSA 0.97 m^2^	Osteosarcoma	450 mg/m^2^	10 d	HeartWare^TM^ LVAD	149 d	Recovery, explant
Lara et al. 2017 [55]	* 1 patient 14 years 51 kg, BSA 1.4 m^2^	AML	150 mg/m^2^	3 months	HeartWare^TM^ LVAD	1 year	Recovery, explant
Bock et al. 2017 [56]	13 patients	Not stated	Not stated	Not stated	Not stated	Not stated	Heart transplant, number of patients not stated
Hope et al. 2020 [57]	2 patients: * 14 years 51 kg, BSA 1.4 m^2^ 7 years 20.6 kg, BSA 0.8 m^2^	AML ×2	150 mg/m^2^, 630 mg/m^2^	3 months	HeartWare^TM^ LVAD	9 months	Recovery, explant ×2
Puri et al. 2021 [58]	1 patient 4 years, 13 kg	Congenital AML	630 mg/m^2^	4 years	HeartWare^TM^ LVAD	6 months	Heart transplant

* Same patient is included in two studies by Lara et al. and Hope et al. ^a^ AML: Acute myeloblastic leukemia, ALL: Acute lymphoblastic leukemia, EWS: Ewing’s sarcoma.

## Data Availability

No new data were created or analyzed in this study. Data sharing is not applicable to this article.
